# COVID-19 highlights health inequalities in individuals from black and minority ethnic backgrounds within the United Kingdom

**DOI:** 10.34172/hpp.2021.15

**Published:** 2021-05-19

**Authors:** Monique Wheatle

**Affiliations:** ^1^Birmingham Medical School, University of Birmingham, Birmingham, UK; ^2^African Caribbean Medical Mentors, London, UK

## Dear Editor,

COVID-19mortality rates in individuals from black African backgrounds are 3.5 times higher, with those from Pakistani and black Caribbean backgrounds increased by 2.7 and 1.7 respectively within the United Kingdom.^1,2^ Whilst research to determine potential biological factors for this increased susceptibility is ongoing, the complex interplay of factors related to socioeconomic status and culture likely also plays a key role. COVID-19 exemplifies the ongoing social and health disparity in individuals of black and minority ethnic (BME) background within the UK, which needs to be addressed.

National Health Service (NHS) ‘frontline’ workers are disproportionately made up of individuals from BME communities, who are less likely to be able to work from home. 64% of the NHS workers who have died from COVID-19, are from BME backgrounds.^3,4^ Up to a 15% increase in individuals suffering from overcrowding has also been reported in these individuals.^1^ As a result, individuals from BME communities may have an increased exposure to COVID-19. BME individuals are also over-represented in groups that are at higher risk of serious complications from the COVID-19 virus.^5^

The incidence of chronic diseases such as cardiovascular disease, hypertension and diabetes, is higher in individuals from BME communities, these are comorbidities known to worsen COVID-19 outcomes.^3^ Wider environmental factors are thought to play important roles in this increased incidence of chronic diseases, including lower socioeconomic status, less access to and poorer experience of healthcare services and cultural identity.^4^ The main health determinant that has an association with these ethnic inequalities is the poorer socio-economic position of BME groups.^6^ For those with mental illness, while service use and referral methods show inequalities, these individuals also have higher unemployment rates, poorer living conditions and poorer health literacy – again showing the impact of poor socioeconomic status on BME individuals.^7-9^ Combined with direct acts of discrimination, increased stress in the workplace, lack of social mobility and poorer experiences of healthcare, these factors contribute to the psyche of these individuals.^1,10^ Even within the NHS, it is well-known that BME staff report higher levels of bullying, harassment and abuse.^3^ It is interesting to consider how feeling like ‘outsiders’ impacts the physical and mental wellbeing of BME individuals – and how the COVID-19 has exacerbated these experiences.^11^

Ongoing health inequalities within the UK are having a considerable impact on the lives and health of BME individuals, these are exemplified by worse outcomes in those with COVID-19 disease. However, these health inequalities are also widely seen elsewhere in the world, including the United States.^12^ The impact of factors discussed above and other country-specific factors throughout the world on health inequalities and COVID-19 outcomes warrants further scrutiny. To implement change, we must begin to address the poor socio-economic status of many BME individuals, as this is a major determinant of ethnic health inequalities. Implementing targets for areas of the country with high health inequality statistics to decrease the inequality gap, specifically targeting factors such as access to healthcare, employment and poverty will be key. Education initiatives, similar to the ‘change for life’ campaigns that promote awareness of health risks and healthy lifestyle may improve health literacy within this population particularly for the health conditions they are more susceptible to.^13^ Work to accurately collect data on ethnicity, and the role of complex social and health factors on the outcomes of COVID-19 for BME individuals is now essential. This will help delineate the complex interplay of these avoidable inequalities and allow a strategy to be created to improve the health of individuals from BME communities.

## Competing interests

None.

## Ethical approval

Not applicable.
